# Limitations of Gliadel Wafers and Strategies for Next-Generation Local Delivery Systems for Glioblastoma

**DOI:** 10.3390/cancers18060907

**Published:** 2026-03-11

**Authors:** Ahmet Kartal, Min J. Kim, Hani Chanbour, Yohannes Tsehay, Safwan Alomari

**Affiliations:** 1 Department of Neurological Surgery, Weill Cornell Medicine, NewYork-Presbyterian Hospital, New York, NY 10065, USA; 2 Harvey W. Cushing Neuro-Oncology Laboratories, Department of Neurological Surgery, Mass General Brigham and Harvard Medical School, Boston, MA 02114, USA; 3 Department of Neurological Surgery, Vanderbilt University, Nashville, TN 37232, USA; 4 Department of Neurological Surgery, Perelman School of Medicine, University of Pennsylvania, Philadelphia, PA 19104, USA; 5 Department of Neurological Surgery, Cleveland Clinic Florida, Weston, FL 33326, USA; 6 Cleveland Clinic Lerner College of Medicine, Case Western Reserve University School of Medicine, Cleveland, OH 44106, USA

**Keywords:** glioblastoma, IDH-wildtype, Gliadel wafers, narrative review, chemotherapy, radiotherapy, immunotherapy, drug development, quality of life (QOL)

## Abstract

**Background**: Local delivery after resection of high-grade glioma, particularly glioblastoma (GBM), aims to increase intratumoral drug exposure while limiting systemic toxicity. The only U.S. Food and Drug Administration (FDA)-approved implantable intracranial chemotherapy is the carmustine (1,3-bis[2-chloroethyl]-1-nitrosourea; BCNU)-impregnated polyanhydride wafer (Gliadel wafer), indicated for newly diagnosed high-grade glioma and recurrent GBM. More than two decades of clinical use and randomized data show that intracavitary chemotherapy is feasible and confers a modest survival benefit in carefully selected patients. Nevertheless, Gliadel wafer has not altered the overall poor prognosis of GBM because of biological resistance to nitrosoureas, constrained brain-parenchymal pharmacokinetics, and device-related adverse effects. **Objective**: The aim is to synthesize clinical and preclinical evidence defining the current limitations of Gliadel wafer and to outline strategies for next-generation local delivery systems, with emphasis on GBM within the broader high-grade glioma setting. **Methods**: A narrative review of randomized and observational clinical studies, pharmacokinetic studies, and preclinical investigations evaluating Gliadel wafer and potential next-generation local delivery systems in GBM and other high-grade gliomas was performed. **Results**: The literature delineates key limitations of Gliadel wafer: short diffusion distances and burst-weighted carmustine release, high tumor cell resistance to carmustine due to heterogeneity, and device-related side effects. Emerging approaches to address these limitations include (i) multidrug systems with synergistic effects against GBM cells; (ii) advanced biomaterials that enable controlled and sustained release; and (iii) targeted agents with minimal off-target effects. Testing newer generations of local drug-delivery systems in more predictive translational models, such as patient-derived organoids and spontaneous large-animal glioma models, is essential to maximize the translatability of preclinical studies to human studies. However, broader adoption of spontaneous large-animal glioma models is constrained by ethical oversight, animal-welfare considerations, cost, and limited availability compared with rodent platforms. **Conclusions**: Next-generation local drug-delivery systems should include multiple synergistic tumor-selective drugs that can be released in a controlled, sustained manner deep into the residual tumor. Preclinical testing of these systems should be conducted in clinically relevant animal models that are more translatable than those used in early Gliadel wafer studies.

## Simple Summary

Glioblastoma is an aggressive brain cancer that almost always returns after surgery. Gliadel wafers are biodegradable disks that contain carmustine and can be placed directly into the surgical cavity to treat newly diagnosed high-grade glioma or recurrent glioblastoma while limiting whole-body exposure. Their benefit has been modest because drug release is front-loaded, tissue penetration is shallow, many tumors are resistant to nitrosoureas, and the implants can cause local complications. In this review, we summarize clinical and laboratory evidence explaining these barriers and outline improved local-delivery designs, including multidrug depots, more conformal biomaterials with slower release, and targeted therapies that may better match glioblastoma biology. We also discuss more predictive translational models that may accelerate the path to clinical testing.

## 1. Introduction

High-grade gliomas remain among the most lethal primary brain tumors in adults, and glioblastoma (GBM) is the most common and aggressive subtype. In the 2021 World Health Organization Classification of Tumors of the Central Nervous System (WHO CNS5), GBM is defined within adult-type diffuse gliomas as “glioblastoma, isocitrate dehydrogenase (IDH)-wildtype” (CNS WHO grade 4), diagnosed by histologic features or by molecular markers such as telomerase reverse transcriptase (TERT) promoter mutation, epidermal growth factor receptor (EGFR) amplification, or combined whole-chromosome 7 gain/10 loss [1]. It remains uniformly lethal despite maximal multimodal therapy. Standard care since 2005 consists of maximal safe resection followed by chemoradiation [2,3]. The median overall survival with contemporary therapy rarely exceeds 15–21 months, underscoring the need for better local control at the resection margin where most recurrences arise [4,5]. Local drug delivery has gained popularity because it bypasses the blood–brain barrier (BBB), potentially increasing drug concentrations in the surgical cavity and the surrounding infiltrating tumor while minimizing systemic exposure [6].

Despite ongoing improvements in surgery, radiotherapy (RT), and systemic treatments, studies on patterns of failure consistently indicate that most recurrences occur within 2 cm of the original tumor site [7]. This suggests that residual cells at the resection margin are not being effectively treated, even when dose constraints are met [7,8]. In this setting, the resection cavity serves as a convenient site for treatment, allowing neurosurgeons to insert implants, catheters, or injectable depots during surgery, with the goal of maintaining high drug levels around the cavity rim for a prolonged time [6,9]. Local approaches, including biodegradable wafers, convection-enhanced delivery (CED), brachytherapy, and in situ-forming depots, are therefore increasingly viewed as complementary to systemic therapy rather than competitors, aiming to intensify treatment where microscopic disease is densest while limiting systemic toxicity [9,10].

Among local strategies, the Gliadel wafer is the first proof-of-concept biodegradable intracavitary chemotherapy implant and remains the only approved intracranial wafer platform currently recognized by the U.S. Food and Drug Administration (FDA) [11,12]. Randomized trials showed modest outcome improvements over placebo in recurrent malignant glioma and newly diagnosed high-grade glioma, with median survival increasing from 5.3 to 7.1 months in recurrent cases, and from 11.6 to 13.9 months in newly diagnosed cases; this corresponds to a 29% relative risk reduction death) [12,13]. These trials predated or only partially overlapped the temozolomide (TMZ) era, and subsequent observational series in the TMZ era have shown heterogeneous results [14,15].

**Figure 1:**
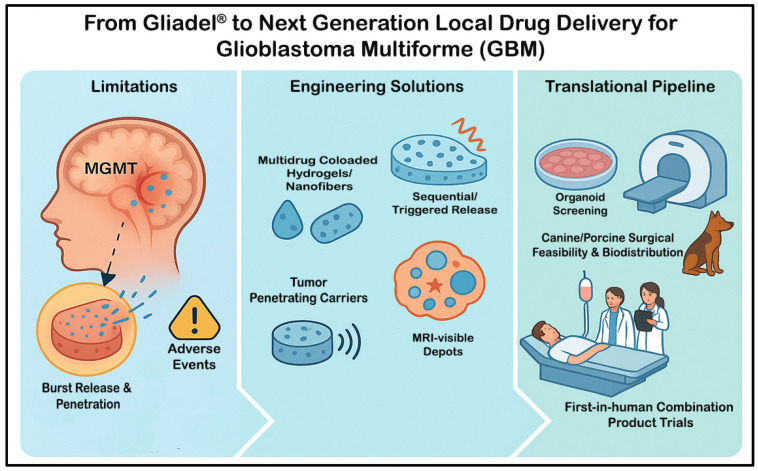
Graphical Summary of the Gliadel Wafer Concept.

This review critically examines the limitations of the Gliadel wafer and proposes new design and translational strategies for next-generation local therapies for GBM. Addressing these limitations can enhance the efficacy and safety of these local drug-delivery systems for patients with GBM (Figure 1).

### Literature Search Strategy

We performed a targeted narrative literature search in PubMed/MEDLINE and Scopus, supplemented by www.ClinicalTrials.gov for ongoing or recently completed trials. The timeframe emphasized January 1990 through October 2025 to capture the pivotal Gliadel randomized trials, subsequent temozolomide-era series, and recent advances in biomaterials and immunotherapy. Search terms included combinations of: “Gliadel”, “carmustine wafer”, “carmustine (BCNU) implant”, “polyanhydride”, “glioblastoma”, “high-grade glioma”, “local delivery”, “intracavitary”, “hydrogel”, “convection-enhanced delivery”, “nanoparticle”, “oncolytic virus”, and “chimeric antigen receptor (CAR) T-cells”. For clinical outcomes, we prioritized randomized trials, meta-analyses, and large observational cohorts; for emerging strategies, we prioritized recent preclinical studies that reported quantitative release kinetics, spatial distribution, safety, and/or in vivo efficacy in orthotopic or resection models. Reference lists of key reviews were also screened to identify additional relevant studies.

## 2. Current Limitations of Gliadel Wafers

Despite establishing the feasibility of local chemotherapy, Gliadel wafers provide only incremental benefit in selected patients because their performance is constrained by tumor biology, pharmacokinetics, and device limitations. On the biology side, single-agent carmustine is blunted by O^6^-methylguanine-DNA methyltransferase (MGMT)-mediated repair and related deoxyribonucleic acid (DNA)-damage responses, so durable control of a heterogeneous, infiltrative GBM is not achieved. Pharmacokinetically, the polifeprosan-20 matrix delivers a short, front-loaded burst of carmustine (1,3-bis[2-chloroethyl]-1-nitrosourea; BCNU) over only a few days, and concentrations fall steeply from the cavity wall, typically reaching just millimeters into the surrounding brain. Practical coverage is further limited by irregular cavity geometry and, when present, cerebrospinal fluid (CSF) communications. Safety profile and adverse events such as edema, seizures, wound-healing problems, CSF leak or infection, and cyst formation can occur. The wafer is brittle and moisture-sensitive (frozen storage and careful placement are required), and, therefore, full dosing is not always achievable. Together, these factors define the ceiling of first-generation wafers and motivate the engineering and translational strategies outlined in Section 3 and Section 4.

### 2.1. Limited Efficacy of Carmustine as a Single Agent

BCNU is a chloroethylnitrosourea that alkylates DNA at the O^6^ position of guanine; the O^6^-chloroethyl adduct can evolve into interstrand cross-links that are highly cytotoxic to tumor cells. [16,17]. The DNA repair protein MGMT removes O^6^ adducts in a single step and is a major determinant of nitrosourea and other O^6^-alkylator resistance. High MGMT activity blunts drug effect, whereas MGMT promoter methylation correlates with greater alkylator responsiveness [18,19]. Mismatch repair and broader DNA damage responses further shape sensitivity to nitrosoureas and TMZ, enabling rapid selection of resistant clones under treatment pressure [17].

Across pivotal randomized trials, the survival gains with Gliadel wafer were modest, typically weeks to a few months, underscoring that single-agent BCNU cannot control a heterogeneous, often MGMT-proficient infiltrative tumor [13,20]. In addition, carmustine has not been shown to have a significant therapeutic effect on GBM stem cells, which are believed to drive tumor recurrence [21]. Importantly, the magnitude of benefit is shaped by patient selection (extent of resection and residual tumor burden), tumor biology (e.g., MGMT status and intra-tumoral heterogeneity), and the diffusion-limited treatment field created by an intracavitary single-agent alkylator [14,15].

In the TMZ era, benefit appears most plausible in well-selected patients—for example, those with near-gross-total resection. Yet contemporary series report heterogeneous results and are vulnerable to confounding by the extent of resection, MGMT status, and adjuvant treatment intensity [14,15,22,23,24]. In practice, guidelines generally frame carmustine wafers as an optional adjunct rather than a routine standard of care, and real-world uptake in Europe is variable due to modest incremental efficacy, added operative risk (edema/seizures, wound complications), logistical constraints, and reimbursement/cost considerations [25,26]. For this reason, some guidelines note limited routine use in Europe [27,28].

At the cellular level, GBM stem-like cells (GSCs) and other treatment-tolerant subpopulations exhibit high DNA-repair capacity, quiescence, and metabolic plasticity, which collectively reduce their sensitivity to BCNU and TMZ [29,30]. These cells often reside at the infiltrative margin, around perivascular niches, and along white-matter tracts—regions that extend beyond the short diffusion radius of carmustine from the cavity wall [31,32]. Heterogeneous transcriptional programs (e.g., proneural, classical, mesenchymal) and dynamic phenotypic switching under treatment pressure further argue against relying on a single alkylator, and instead support rational local combinations that simultaneously target DNA damage response, invasion, and stem-cell survival pathways [33,34].

Taken together, there is a consistent theme: even when relative risk reductions appear meaningful, the absolute survival gains are often small and vary across cohorts. This heterogeneity likely reflects differences in the extent of resection, cavity geometry, adjuvant therapy (including TMZ), and tumor biology (e.g., MGMT and proliferative index), and it underpins why many centers reserve Gliadel for highly selected surgical candidates rather than adopting it routinely [14,15,24].

### 2.2. Pharmacokinetic Constraints

The polyanhydride matrix (polifeprosan 20) hydrolyzes after implantation, producing an early burst of carmustine followed by a rapid decline in release; experimental work shows that most of the drug elutes over several days, while the polymer remnants persist for weeks [35]. Human pharmacokinetics mirror this behavior: systemic BCNU is detectable for about 24 h, with a maximal blood concentration occurring about 3 h after placement, supporting the evidence of limited, short-lived output from the implant [36,37]. Drug levels decrease rapidly as they move away from the cavity wall, so significant drug concentrations are confined to a few millimeters into the surrounding tissue. This leaves any deeper invasive disease untreated. Both computer simulations and animal studies support the existence of this sharp concentration gradient [38,39]. Reports on diffusion distances differ by model; however, even the more optimistic studies suggest typical penetration is in the millimeter range [20]. Reaching infiltrative tumor cells up to ~2 cm from the resection margin is unlikely to be achievable by diffusion alone. 

Among existing locoregional approaches, CED is the most direct strategy to extend drug distribution via bulk interstitial flow, particularly when paired with reflux-resistant catheters and image guidance [38]. Other concepts under exploration include distributing multiple small depots along the infiltrative rim, using actively migrating cellular carriers, and combining implants with repeatable catheter-based infusion or externally triggered release. Nevertheless, achieving reliable and safe 2 cm coverage in the human brain remains a central translational hurdle for all local delivery platforms.

**Figure 2:**
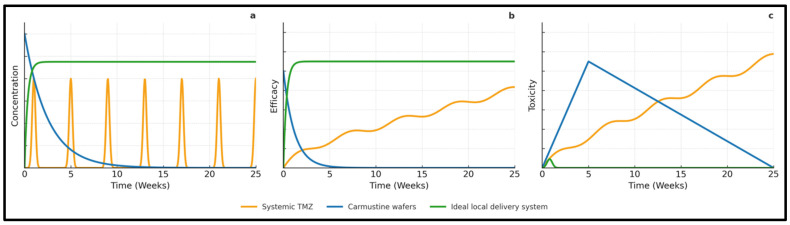
Schematic Comparison of Systemic Temozolomide, Carmustine Wafers, and an Ideal Local Drug Delivery System. Conceptual comparison of drug exposure, therapeutic effect, and toxicity for standard systemic temozolomide (TMZ) given per the Stupp regimen, carmustine implants, and a proposed optimized local delivery platform. (**a**) Local administration can generate substantially higher drug levels at the target site than systemic TMZ, whose dose is constrained by whole-body exposure; an optimized system would provide prolonged release with approximately zero- or first-order kinetics. (**b**) Maintaining elevated local concentrations over time with such a device is expected to support sustained tumor control and reduce the likelihood of recurrence. (**c**) Adverse effects associated with the mechanical stiffness of carmustine wafers could be mitigated by designing softer, more compliant materials, while systemic toxicity continues to limit further intensification of TMZ-based regimens. Reproduced with permission from Tabet et al. [41], Advanced healthcare materials; published by Weinheim: Wiley-VCH, 2019.

In real-world practice, these pharmacokinetic challenges are further complicated by the resection cavity’s shape [9,20]. Surgeons often need to cut wafers into smaller pieces, leave some surfaces exposed, or avoid placing wafers near sulci, ventricles, or cisterns to minimize migration and cerebrospinal fluid obstruction [20,25]. Consequently, only a portion of the peri-cavity surface contacts the drug-eluting polymer directly, creating a steep concentration gradient [20,40]. This means that cells just a few millimeters away often receive subtherapeutic doses, which explains why tumors tend to recur just beyond the expected diffusion zone [7,40]. Developing more uniform and conformal depots remains an important goal for future treatments, and these conceptual relationships between drug exposure, efficacy, and toxicity for systemic TMZ, Gliadel wafers, and an idealized local delivery platform are summarized in Figure 2.

### 2.3. Safety, Adverse Effects, and Handling Constraints

Post-implant issues include peri-cavitary edema, seizures, wound-healing problems, CSF leak/meningitis, cyst formation, and intracranial hypertension. The FDA label reports seizures in up to 37% of patients with recurrent disease and cautions about edema and wound-healing risks that warrant close monitoring [37]. Case series and reviews report instances of peri-cavitary edema that are severe enough to exert pressure on surrounding structures, sometimes leading to hydrocephalus or displacement of wafers, especially when the cavities are connected to CSF pathways [42,43]. Although systemic exposure is minimal and temporary, blood levels can be detected for approximately 24 h following placement. As a result, the mutagenic and teratogenic risks associated with carmustine necessitate precautions related to contraception and breastfeeding [37].

Each wafer contains 7.7 mg of carmustine embedded in polifeprosan-20, which is a polymer composed of bis(p-carboxyphenoxy) propane and sebacic acid in a 20:80 ratio. It is recommended to use up to eight wafers to cover the affected area [37]. Labels and technical sheets specify frozen storage (≤−20 °C) and careful intraoperative handling due to moisture sensitivity and fragility; in practice, irregular cavity geometry often prevents achieving the full eight-wafer dose [37].

Meta-analyses and large retrospective series confirm that these device-related toxicities are not rare, particularly when wafers are combined with standard postoperative chemoradiation [44,45]. Higher rates of cerebral edema, wound dehiscence, and intracranial infection have been reported compared with historical controls, and some series describe cystic degeneration of the cavity that can necessitate re-operation or long-term CSF diversion [46,47]. In patients with limited performance status or multiple comorbidities, the clinical impact of such complications may outweigh the modest survival benefit [13,45]. Consequently, many centers limit Gliadel use to carefully selected patients—typically those with good functional status (e.g., Karnofsky ≥ 70), supratentorial tumors amenable to near-gross-total resection, and cavities without direct communication to the ventricular system or basal cisterns [13,25]. Intraoperatively, best practices include gentle placement under direct visualization, avoiding multilayer stacking in narrow recesses, and striving for watertight dural closure to minimize CSF leak and wafer migration [20,25]. Even with meticulous technique, however, the wafer’s brittleness, fixed dimensions, and moisture sensitivity make it challenging to achieve uniform coverage of complex cavities, underscoring the need for more conformable, robust local delivery systems [6,48].

## 3. Emerging Strategies to Overcome Current Limitations

Local therapy for GBM is evolving from single-agent, fast-eluting wafers toward combination depots and smarter materials that relatively match the biology and geometry of the disease. The most promising directions pair rational drug combinations with delivery matrices that release for months, conform to complex cavities, and spread the drug deeper into the peri-resection cavity parenchyma.

### 3.1. Multidrug Local Delivery Systems

Given GBM’s heterogeneity, stem cells, and rapid adaptive resistance, local drug combinations are more likely to succeed than replicating the first-generation single-agent approach. Numerous drugs have been discovered or repurposed to target GBM cells and have shown significant therapeutic efficacy in in vitro and in vivo studies. However, the ideal combination to eradicate the heterogeneous cell populations in GBM remains to be identified [49].

In orthotopic models, co-loaded polymer implants have outperformed single-drug wafers—for example, TMZ and BCNU released from a single biodegradable wafer produced stronger tumor control than either agent alone, establishing feasibility for intracavitary combination chemotherapy [50]. Similar results have been shown with TMZ and paclitaxel packaged in a photopolymerizable hydrogel or pellets, which provided sustained release, better conformity to complex margins, and improved survival compared with one-drug depots [51,52,53,54]. Together, these data suggest that, when used locally, combinations can complement each other mechanistically without increasing surgical morbidity or systemic toxicity [20].

A key challenge is carefully choosing which drugs to combine locally rather than empirically co-loading multiple cytotoxics [55,56]. Modern genomic and transcriptomic profiling reveals substantial inter- and intra-tumoral variation in pathways such as receptor tyrosine kinase/rat sarcoma/phosphoinositide 3-kinase (RTK/RAS/PI3K), cell-cycle regulation, and DNA repair, suggesting that ideal combinations will pair a DNA-damaging backbone (e.g., alkylators or topoisomerase inhibitors) with agents targeting compensatory survival pathways, invasion, or stem-cell maintenance [57,58]. Local depots also offer an opportunity to co-deliver agents that are poorly tolerated systemically—such as high-dose alkylators or radiosensitizers—while keeping systemic exposure low, potentially widening the therapeutic window when combined with RT and systemic TMZ [20,59].

In practice, pathway prioritization for local combination studies can be made more explicit by aligning the payload with (i) recurrently altered GBM nodes, such as RTK/RAS/PI3K signaling, cell-cycle regulators such as cyclin-dependent kinases 4 and 6 (CDK4/6), and DNA-damage response/repair; (ii) known resistance mechanisms to alkylators, such as MGMT-mediated repair and adaptive survival signaling; and (iii) drug properties that make systemic use ineffective or intolerable, including poor BBB penetration and dose-limiting hematologic toxicity [57,58]. This framework supports pairing a DNA-damaging backbone with locally delivered inhibitors of compensatory survival pathways, including PI3K/mammalian target of rapamycin (mTOR) and mitogen-activated protein kinase (MAPK), DNA repair targets such as poly(ADP-ribose) polymerase (PARP), or stem-cell maintenance programs, while leveraging local delivery to explore combinations that would be unsafe at systemic exposures.

### 3.2. Engineering-Enhanced Wafer Systems

Advances in materials engineering provide novel approaches to address the pharmacokinetic and spatial constraints of current wafer systems [60]. Specifically, hydrogels and nanofiber meshes can be precisely tuned for prolonged drug release and sequential dosing. Layered or core–sheath meshes, for instance, facilitate the immediate delivery of a radiosensitizer followed by extended chemotherapy. Furthermore, conformal patches enhance the interfacial surface area for drug diffusion without increasing intracranial pressure [61,62,63].

With respect to safety, a key differentiator of many injectable hydrogels is their mechanical compliance and conformal filling of irregular cavities: softer, tissue-matched materials may reduce focal mass effect and mechanical irritation at the cavity wall (a proposed contributor to peri-cavitary edema and seizures with rigid wafers). Hydrogels also allow finer control over burst release and local peak concentrations, potentially smoothing early drug spikes that can exacerbate inflammatory edema while still maintaining high local exposure over time [64,65]. That said, any intracranial implant or depot can provoke edema, and translation will require systematic safety monitoring (steroids, imaging, and seizure surveillance) alongside pharmacokinetic validation.

Regarding the “zero-order” release concept in Figure 2, several depot designs (including in situ–forming hydrogels and erosion-controlled matrices) have demonstrated approximately linear, sustained release kinetics in vitro over clinically relevant timescales; however, true zero-order behavior in vivo is uncommon and can be perturbed by swelling, enzymatic degradation, and CSF exchange [64,65].

Beyond sustained release profiles, in situ-forming, tissue-adhesive hydrogels improve retention on irregular walls and have delivered therapeutic proteins and antibodies in primary human GBM models, supporting their use as conformal depots after resection. To push the drug beyond the millimeter-scale rim, carriers can be equipped with tumor-penetrating ligands, such as arginine-glycine-aspartic acid (RGD) or internalizing RGD (iRGD) peptides, or built using layer-by-layer (LbL) surface chemistry; both approaches increase brain-tissue distribution and glioma-cell uptake in vivo, with LbL nanoparticles also enabling magnetic resonance imaging (MRI) visualization of local spread [66,67]. Beyond nanoparticle mapping, implantable imaging-visible depots have also advanced; for instance, MRI-monitored hydrogels have been tracked longitudinally in the brain, opening the door to noninvasive placement checks and dose–response readouts in early trials [68]. In addition, triggerable systems (e.g., ultrasound/sonodynamic-responsive formulations) are being developed to boost release or add orthogonal cytotoxic mechanisms while preserving intracranial biocompatibility [69].

Beyond simply prolonging drug release, advanced biomaterials can be designed to actively influence the surrounding tissue environment. Hydrogels with customizable stiffness, porosity, and degradation rates can be tailored to facilitate deeper tissue penetration or to align with nearby blood vessels [48,70]. Electrospun meshes can be layered or patterned to establish spatial drug gradients that better target infiltrative tumor margins [62]. Thermoresponsive nanocomposite hydrogels, injected as liquids and solidifying at body temperature, can create soft depots lining surgical cavities, as shown in orthotopic GBM models, where these hydrogels provide extended intracortical drug release with minimal initial burst [64,65]. Emerging stimuli-responsive depots can also be activated by external triggers, such as magnetic fields or focused ultrasound, to control drug release on demand [71,72]. For instance, hydrogels containing magnetic nanoparticles can generate mild heat under alternating magnetic fields, speeding up drug diffusion and improving tissue penetration [71]. Ultrasound-responsive systems may allow noninvasive enhancements of local therapy at specific times [72,73]. Additionally, nano-engineered hydrogels that co-deliver chemotherapeutic drugs with immunomodulators or metabolic agents have been shown to remodel the immune and stromal microenvironment, increasing T-cell infiltration and reducing tumor recurrence in preclinical GBM models [70,74]. These advances suggest that future drug-delivery wafers could become programmable, visible reservoirs capable of controlled drug release, rather than passive, degrading disks [6,74].

### 3.3. Targeted Therapeutics

In addition to cytotoxic drugs, local delivery systems can provide targeted therapies like oncolytic viruses and small-molecule or antibody treatments. Oncolytic viruses preferentially replicate in cancer cells, sparing normal cells, and induce direct oncolysis and promote immunogenic cell death. A phase I study of DNX-2401 (Delta-24-RGD), an oncolytic adenovirus, reported that among 37 patients with recurrent malignant glioma (including GBM and IDH-wildtype), 20% survived longer than 3 years and 12% achieved greater than a 95% reduction in tumor enhancement [75]. Another phase II trial using intratumoral oncolytic herpes virus G47Δ in 19 adult patients with residual or recurrent supratentorial GBM demonstrated a 1-year survival rate of 84.2% with a median overall survival of 20.2 months from therapy initiation [76]. Finally, in a multicenter phase 1/2 study combining oncolytic DNX-2401 virotherapy with pembrolizumab, no dose-limiting toxicities were observed, and the 12-month overall survival was 52.7%, compared with only 20% in the control group [77].

Local or intracavitary administration is particularly appealing for biological agents whose systemic use is limited by toxicity, immunogenicity, or poor BBB penetration [78,79]. Catheter-based CED has already enabled focal infusion of recombinant toxins, radiolabeled antibodies, and immune agonists deep within the brain, and similar pharmacokinetic principles can be harnessed by biodegradable depots positioned along the cavity wall [80,81]. By co-localizing oncolytic viruses, cytokines, or checkpoint modulators with regions of greatest residual tumor burden, future platforms may amplify intratumoral immune activation while keeping systemic exposure low and reducing the risk of off-target organ toxicity [74,82].

Small-molecule/antibody conjugates for local delivery can be engineered as locoregional depots to release molecularly selective payloads—including small-molecule conjugates, antibody fragments, or peptide-decorated nanoparticles—so that uptake is driven by tumor-specific ligands while exposure remains concentrated around the cavity. Integrin-targeted designs (e.g., RGD/iRGD) improve cellular entry and tissue spread in GBM models and can be embedded in hydrogels to sustain release and broaden coverage at the infiltrative edge [83,84]. CD44-directed systems, often built on hyaluronic acid (HA) backbones, exhibit preferential uptake by GBM cells over normal glia and enhanced antitumor activity in orthotopic studies, making hyaluronic acid-nanoparticle (HA-NP)/hydrogel composites attractive for postoperative intracavitary delivery [85,86]. Hydrogel–nanoparticle matrices have already demonstrated feasibility in in-cavity placement and survival gains in rodents, supporting the platform concept for conjugated or ligand-guided agents after resection [10,64]. Furthermore, these targeted biomaterial depots could be synergistically combined with other locoregional modalities, such as laser interstitial thermal therapy (LITT)—a minimally invasive cytoreductive approach—to maximize local disease control in selected settings [87].

However, translating treatments to patients still poses challenges: for instance, the EGFR-targeted antibody-drug conjugate (ADC) depatuxizumab mafodotin (ABT-414) did not improve overall survival in a phase 3 trial with newly diagnosed glioblastoma patients, despite a strong biological rationale. This highlights the importance of combining smarter targeting with appropriate local delivery and patient selection [88]. Recent advances in engineering (e.g., micro-mesh and conformal hydrogel depots that deliver nanoparticles or antibody fragments) aim to provide sustained, spatially uniform release compatible with neurosurgical workflows, but clinical validation in GBM remains pending [89].

Another emerging direction is cell-based local immunotherapy. Early clinical experience with dual-targeting CAR T-cells directed against antigens such as EGFR and interleukin-13 receptor alpha 2 (IL13Rα2) has shown that intracavitary or intraventricular dosing is feasible, and that organoid-based avatars can help interpret response patterns and toxicity [90,91]. Embedding CAR T-cells, engineered macrophages, or dendritic-cell vaccines within supportive hydrogels or porous scaffolds is being explored preclinically to prolong cell persistence, guide their migration into the infiltrative rim, and shield them from hostile microenvironmental cues [92]. Although still at a conceptual stage, these strategies highlight how intracavitary depots could eventually function as multifunctional “immune niches” that release drugs, living effector cells, and even diagnostic reporters in a coordinated fashion. Embedding CAR T-cells (or other immune effectors such as macrophages or dendritic-cell vaccines) within supportive hydrogels or porous scaffolds is proposed to improve local efficacy through several non-mutually exclusive mechanisms: (i) physical retention and broad surface coverage of the resection cavity to reduce immediate wash-out into CSF; (ii) provision of a temporary extracellular-matrix-like niche that sustains viability and enables gradual cell egress into the infiltrative rim; and (iii) co-delivery of immunomodulatory cues (e.g., cytokines/chemokines or checkpoint blockade) to counteract local immunosuppression at the wound-tumor interface [92,93,94]. In the intracranial setting, these designs must be balanced against unique safety risks: an exuberant local immune response can precipitate edema, mass effect, seizures, and elevated intracranial pressure, and therefore requires dose control, careful imaging surveillance, and clear management algorithms (e.g., corticosteroids or cytokine-directed rescue in severe cases).

### 3.4. Comparative Readiness and Translational Barriers

To move beyond a descriptive catalog of technologies, it is useful to contrast local delivery strategies by (i) spatial coverage of the infiltrative rim, (ii) pharmacologic flexibility (single-agent vs. multidrug/biologics), and (iii) clinical readiness. In broad terms, biomaterial depots (hydrogels, electrospun meshes, and implantable reservoirs) are relatively mature from a materials and manufacturing standpoint, but they must still demonstrate reliable distribution beyond the cavity wall and predictable in vivo release [6]. By contrast, oncolytic viruses have progressed to multiple early-phase trials and can spread beyond the initial injection site, yet they face distinct hurdles, including vector immunogenicity, control of biodistribution, and complex Good Manufacturing Practice (GMP) manufacturing [79]. Cell therapies such as CAR T-cells offer potent, adaptable cytotoxicity but remain challenged by antigen heterogeneity, immunosuppressive microenvironments, neurotoxicity risks, and the cost-intensive, individualized manufacturing required [79]. Regulatory pathways also differ: many intracavitary depots are “combination products” (device + drug) that require integrated validation of biocompatibility, sterility, and release profiles, whereas viral and cell therapies require extensive characterization of replication competence, shedding, persistence, and off-target activity [79]. Across all approaches, translation is limited not only by efficacy signals but by practical implementation barriers—operating-room workflow, reproducibility of placement, perioperative safety monitoring, and health-economic feasibility [79].

**Figure 3:**
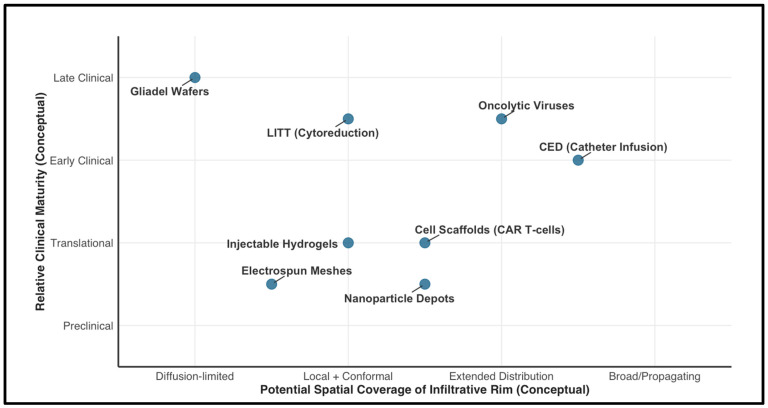
Conceptual and Illustrative Positioning of Representative Locoregional Strategies by Relative Clinical Maturity and Potential Spatial Coverage of the Infiltrative Rim (not for Quantitative Purposes). ***Abbreviations:*** CAR, chimeric antigen receptor; CED, convection-enhanced delivery; LITT, laser interstitial thermal therapy.

Figure 3 presents an illustrative map of representative locoregional strategies, positioned by relative clinical maturity and potential spatial coverage of the infiltrative rim; it is intended for conceptual guidance only and not for quantitative interpretation. A comparative synopsis of these emerging locoregional strategies is provided in Table 1.

## 4. Translational Path: Beyond Traditional Preclinical Models

The Gliadel wafer was approved many years ago for local chemotherapy treatment of GBM and served as an early proof of concept for delivering drugs directly into the brain. However, its preclinical testing used 9 L gliosarcoma cells in rats, a model now known to poorly predict how human GBM behaves. These limitations include limited tumor diversity, unnatural growth patterns, and failure to mimic interactions between the tumor and the immune system, all of which contribute to the high failure rate when translating findings to clinical settings [95,96]. Therefore, next-generation local drug-delivery platforms require preclinical models that better reflect the molecular, genetic, and immunological context of human GBM. Key translational models and their specific contributions to local-delivery development are summarized in Table 2.

### 4.1. Efficacy Preclinical Studies

Patient-derived xenografts (PDXs) and GBM organoids (GBOs) have emerged as robust tools for bridging this gap [97]. Derived from patient tumor tissue or short-term cultured cells, these models preserve tumor heterogeneity and molecular characteristics, allowing realistic assessment of therapeutic efficacy [98]. Implantation in immunocompetent mice under short-term immunosuppression enables evaluation of therapies in the context of an intact immune system, capturing pro- and anti-tumor immune responses [99].

A practical limitation of current GBM organoid platforms is the absence of a functional vascular system and bulk interstitial flow, which means that drug distribution in vitro is primarily diffusion-limited and may underrepresent transport barriers (or advantages) seen in the living brain [100]. For local-delivery studies, this can be partially addressed by (i) using microfluidic “organ-on-chip” perfusion systems, (ii) incorporating endothelial/stromal co-cultures to promote vascular-like networks, or (iii) applying depots directly to organoid or organotypic slice surfaces to quantify penetration gradients [101]. Ultimately, organoids are best viewed as complementary screening models, and spatial-distribution claims for local delivery should be validated in orthotopic/resection models where CSF dynamics and tissue architecture are preserved [79].

Beyond serving as generic efficacy platforms, PDX and organoid systems are increasingly being used in “functional precision medicine” workflows in which each patient’s tumor generates a small biobank of models [102,103]. These patient-derived models can be screened ex vivo against panels of systemic and locally deliverable agents, including potential intracavitary drug combinations, with results feeding back into clinical decision-making within clinically relevant time frames [102,104]. For local drug-delivery research, such pipelines enable evaluation of which drug cocktails (e.g., alkylators plus targeted agents or immunomodulators) are most active against an individual tumor before embedding them into wafers or hydrogels, thereby de-risking early-phase trials and aligning intracavitary therapy with patient-specific vulnerabilities [6,103].

To enhance clinical relevance, standardized rodent tumor resection systems have been developed [105]. These minimally invasive devices combine suction and a precision cutting blade to remove tumors through the original burr hole, reducing operative time, blood loss, and postoperative morbidity, while preserving tissue viability for downstream analyses. This method aids in researching perioperative biology and intra-cavity drug delivery, providing a more accurate representation of how surgery affects treatment responses [105].

In translation, more predictive biological models must be matched to clinically meaningful endpoints. Incorporating standardized patient-reported outcomes (PROs) and neurocognitive testing into trials of local therapies will be essential to ensure that modest survival gains are not offset by deterioration in function or quality of life (QOL) [106,107]. Recent work shows that even when overall survival for GBM is poor, carefully delivered chemoradiotherapy can still improve specific dimensions of health-related QOL, such as emotional and social functioning, emphasizing the importance of capturing patient-centered benefits [108,109]. Contemporary neuro-oncology frameworks now recognize perceived cognition, communication difficulty, seizures, physical functioning, and symptomatic adverse events as core PRO domains that should accompany radiographic and survival endpoints in brain-tumor trials [106,110]. Local delivery platforms that prolong steroid dependence, increase seizure burden, or impair wound healing could negatively affect these domains even if progression-free survival improves, whereas technologies that reduce hospitalizations or allow earlier initiation of systemic therapy may offer net QOL advantages that deserve equal weight in clinical decision-making [110,111].

### 4.2. Safety and Biodistribution Preclinical Studies

For safety and pharmacokinetic considerations, small animals are insufficient because their brain size, tissue structure, and drug distribution differ significantly from those of humans. Large-animal models, such as dogs, allow researchers to optimize implant size, drug payload, and release rates under conditions that more closely mimic human physiology. By combining efficacy studies using PDX or GBO models, including those with resected tumors, with large-animal safety and pharmacokinetic testing, we can create a comprehensive, translational framework. This approach enhances the evaluation of advanced local drug-delivery systems, bringing us closer to effective human treatments. Spontaneous gliomas in pet dogs have become particularly valuable in this context because they more closely recapitulate the size, microenvironment, and clinical course of human GBM than induced rodent tumors [112,113]. In a landmark study, CED of irinotecan (CPT-11)-loaded liposomes into canine gliomas achieved robust intratumoral distribution on real-time MRI and produced volumetric responses that correlated with the volume of distribution, validating both the model and the delivery technique [112]. Later work using biodegradable TMZ-loaded microcylinders implanted into partially resected canine tumors demonstrated the feasibility of postsurgical intracavitary chemotherapy and yielded detailed pharmacokinetic and safety data that would be difficult to obtain in human subjects [114]. These naturally occurring canine models also capture clinically relevant variables such as tumor-related seizures, chronic steroid use, and heterogeneous supportive care—factors that influence edema, wound healing, and device performance [113]. As such, they are well-suited to identify device-specific complications (e.g., cyst formation, edema around depots, catheter malfunction) before first-in-human trials [112,114]. Integrating large-animal studies with rodent models and patient-derived organoids can create a multi-tiered translational pipeline in which candidate local therapies are first screened for efficacy, then stress-tested for distribution and safety in realistic brains, and finally advanced into adaptive early-phase clinical trials [102,112].

Ethical considerations are a major barrier to broader incorporation of large-animal glioma models in the drug-development pipeline. Induced intracranial tumor models in large mammals raise a higher ethical burden because they require creating a lethal disease in sentient animals with longer lifespans and greater welfare needs, and they demand substantial analgesia, monitoring, and humane-endpoint planning [115]. By contrast, many “spontaneous” canine glioma studies enroll client-owned pet dogs with naturally occurring tumors under veterinary ethics oversight and informed owner consent, often with therapeutic intent [116]. Even in this setting, costs, limited case numbers, heterogeneous tumor biology, and the need for specialized imaging/neurosurgical infrastructure constrain scalability relative to rodent platforms [117].

Early-phase human studies also offer an opportunity to refine regulatory science around intracavitary treatments [118,119]. Unlike purely systemic drugs, local depots and CED systems are combination products whose performance depends as much on surgical workflow, catheter positioning, and imaging guidance as on the pharmacology of the payload itself [9,119]. Harmonized reporting standards for implant placement, intra-operative complications, and longitudinal imaging changes—analogous to existing Response Assessment in Neuro-Oncology (RANO) and Response Assessment in Neuro-Oncology Patient-Reported Outcomes (RANO-PRO) initiatives for outcome assessment—would facilitate cross-platform comparison and accelerate the identification of best practices [106,110]. At the same time, phase I–II trials should incorporate detailed pharmacokinetic and pharmacodynamic sampling from re-resected tissue whenever ethically feasible, as exemplified by chronic topotecan CED studies that correlated intratumoral exposure with changes in proliferation and metabolism [118,119]. Such data help clarify whether disappointing clinical results reflect inadequate drug delivery, intrinsic resistance, or off-target toxicity, thereby guiding rational iteration of both drug combinations and device architecture [118,119].

## 5. Conclusions

The use of Gliadel wafers after tumor resection has shown that intracavitary chemotherapy is feasible and can offer modest survival benefits, but overall outcomes in GBM remain poor due to the limited efficacy of single-agent carmustine, short and localized drug exposure, and device-related complications. Next-generation locoregional systems must therefore go beyond “more drug for longer” by combining multiple or targeted agents to overcome resistance, enabling controlled, sustained, and homogeneous drug delivery, and minimizing toxicity. Ideally, intracavitary platforms should accommodate multidrug payloads with independently tunable release, conform to irregular cavity geometries without causing mass effect, degrade predictably while maintaining good biocompatibility in blood and CSF, and remain compatible with repeat surgery, re-irradiation, and advanced imaging. Features like MRI visibility and externally triggered release could turn them into true theranostic devices. Progress will depend on integrating robust preclinical testing (patient-derived xenografts, organoids, large-animal models) with adaptive, biomarker-driven clinical trials in well-selected surgical candidates, using correlative measures such as cavity-wall biopsies, advanced MRI, and circulating tumor DNA to link local pharmacokinetics to biological and clinical effects. Ultimately, improving Gliadel will require the coordinated advances in materials science, rational drug combinations, rigorous translational modeling, and carefully designed clinical implementation.

## Figures and Tables

**Table 1:** Comparative Analysis of Emerging Local-Delivery Strategies Proposed to Overcome Gliadel Wafer Limitations in Glioblastoma.

Emerging Strategy	Comparative Analysis
Multidrug cytotoxic depots • **Biodegradable co-loaded wafers** • **Hydrogels/pellets with ≥2 drugs**	Address: single-agent resistance/heterogeneity; increase local multi-mechanism cytotoxicity while keeping systemic exposure low. Advantages: enable BBB-limited or systemically toxic agents locally. Challenges: rational selection of combination therapies, drug–matrix compatibility, local edema/toxicity; still largely diffusion-limited unless paired with penetration enhancers. Maturity: mainly preclinical orthotopic/resection models; early combination experiences.
Resistance/sensitization combinations • **MGMT depletion with O^6^-benzylguanine + BCNU implant** • **Local modifiers/radiosensitizers with RT/TMZ**	Address: MGMT/DNA-repair driven alkylator resistance; leverage synergy with radiotherapy/standard TMZ. Advantages: mechanism-driven sensitization may increase efficacy without higher systemic alkylator doses. Challenges: systemic toxicity for some sensitizers, complex scheduling, and limited/heterogeneous clinical efficacy signals.
Conformal in situ–forming depots • **Injectable/tissue-adhesive/thermoresponsive hydrogels** • **Nanomedicine-loaded hydrogels**	Address: cavity geometry (conformal coverage), burst release, and mechanical irritation of rigid wafers. Advantages: tissue-matched compliance; tunable degradation with sustained (near-linear) release; compatible with multidrug payloads and immunomodulators. Challenges: in vivo kinetics can deviate from in vitro (swelling/enzymes/CSF exchange); sterilization/GMP scale-up; intracranial edema/ICP monitoring.
Programmable meshes/patches • **Electrospun or multilayer core–sheath membranes** • **Sequential drug-release designs**	Address: need for longer and programmable release (including sequential dosing) and improved surface contact. Advantages: staged therapy (e.g., early radiosensitizer then prolonged chemo); robust handling; potential for engineered gradients at the infiltrative rim. Challenges: achieving conformality in complex cavities; diffusion-limited depth without adjunct distribution strategies; manufacturing reproducibility.
Nanoparticle-mediated targeting systems • **Layer-by-layer functionalized nanoparticles** • **RGD/iRGD tumor-penetrating ligands** • **HA–CD44 targeted systems**	Address: short diffusion radius and non-selective exposure by improving peri-cavity penetration and GBM-cell uptake. Advantages: modular payloads (poorly soluble drugs, biologics); can be embedded in hydrogels/meshes; potential to reduce off-target effects. Challenges: heterogeneous/variable target expression and phenotypic switching; negative systemic EGFR-targeted ADC trial underscores selection challenges; nanoparticle toxicity/clearance; complex CMC and “combination product” regulation.
Imaging-visible/theranostic platforms • **MRI-monitored hydrogels** • **MRI-trackable nanoformulations/image-guided distribution**	Address: uncertainty in placement and in vivo distribution; enable exposure verification and longitudinal monitoring. Advantages: noninvasive QA of depot integrity/coverage; support adaptive early-phase trial designs. Challenges: imaging signal may not perfectly map drug concentrations; added agents must be safe; increased regulatory complexity.
Stimuli-responsive/triggerable systems • **Magnetic nanoparticle hyperthermia-assisted depots** • **Ultrasound-responsive hydrogels; sonodynamic add-ons**	Address: fixed kinetics of passive depots and limited spatial effect by enabling on-demand release boosts or orthogonal cytotoxic mechanisms. Advantages: temporal control; potential synergy with immunotherapy and checkpoint blockade. Challenges: device hardware, dosimetry, and safety (heating, BBB effects, edema/ICP); reproducibility in the human brain.
Implantable microdevices/reservoirs • **μMESH-enabled sustained delivery of molecular and nanoformulated drugs**	Address: need for long-duration, predictable delivery and compatibility with diverse formulation types. Advantages: high loading flexibility; sustained release architecture potentially decoupled from polymer erosion. Challenges: neurosurgical workflow and biocompatibility over months; device-related complications; combination-product regulatory pathway.
CED-based distribution strategies • **Reflux-resistant catheter infusion** • **Chronic CED with PD sampling** • **MRI-tracked CED in large brains**	Address: core diffusion limitation by achieving centimeter-scale volume of distribution through bulk interstitial flow. Advantages: adjustable dosing; compatible with large biologics/toxins/radiopharmaceuticals; exposure–response can be quantified. Challenges: catheter placement/reflux, heterogeneous distribution, infection risk, and operational complexity.
Targeted biologics & immunotherapy • **Oncolytic viruses DNX-2401/G47Δ** • **Virotherapy + checkpoint blockade** • **Viral-vector local immunotherapy**	Address: need for tumor-selective oncolysis and immune activation, with potential spread beyond the initial delivery zone. Advantages: early-phase clinical signals of durable responses in subsets; synergy with checkpoint inhibitors while limiting systemic exposure. Challenges: immunogenicity and control of biodistribution/shedding; inflammatory edema; GMP manufacturing and patient selection.
Local cell therapies • **Intrathecal/intracavitary bivalent CAR T-cells** • **Hydrogel/fibrin/thermoreversible cell depots** • **Organoid “avatars”**	Address: antigen heterogeneity and limited persistence by maximizing local exposure/retention, with scaffold-guided egress into the infiltrative rim. Advantages: programmable cytotoxicity; local dosing feasible; scaffolds may improve persistence and reduce immediate CSF washout. Challenges: neurotoxicity/edema and seizure risk; antigen escape; individualized manufacturing/cost; require strict dose control and management algorithms.

***Abbreviations:*** ADC, antibody–drug conjugate; BBB, blood–brain barrier; BCNU, carmustine (1,3-bis(2-chloroethyl)-1-nitrosourea); CAR, chimeric antigen receptor; CD44, cluster of differentiation 44; CED, convection-enhanced delivery; CMC, chemistry, manufacturing, and controls; CSF, cerebrospinal fluid; DNA, deoxyribonucleic acid; DNX-2401, Delta-24-RGD oncolytic adenovirus; EGFR, epidermal growth factor receptor; G47Δ, oncolytic herpes simplex virus (G47Δ); GBM, glioblastoma; GMP, Good Manufacturing Practice; HA, hyaluronic acid; ICP, intracranial pressure; iRGD, internalizing arginine–glycine–aspartic acid tumor-penetrating peptide; MGMT, O^6^-methylguanine-DNA methyltransferase; MRI, magnetic resonance imaging; PD, pharmacodynamic(s); QA, quality assurance; RGD, arginine–glycine–aspartic acid; RT, radiotherapy; TMZ, temozolomide; μMESH, micro-mesh (μMESH-enabled platform).

**Table 2:** Comparative overview of emerging translational strategies.

Emerging Translational Strategy	Contribution to Local-Delivery Development
Patient-derived models **(e.g., PDX, GBM organoids)**	Enable patient-specific efficacy screening and rational payload/combination selection before embedding into depots. Limitations: lack full vascular/CSF dynamics and interstitial flow in vitro; distribution claims still need orthotopic/resection validation.
Microfluidic BBB/organ-on-chip perfusion platforms	Quantify transport/trafficking of BBB-penetrant or nano-enabled carriers under controlled flow. Limitations: simplified physiology; complements but does not replace in vivo safety/distribution testing.
Orthotopic rodent models with standardized resection tools	Recreate post-resection cavity and wound–tumor interface relevant to intracavitary implants; support iterative testing of conformality, early safety, and local PK/PD. Limitations: rodent scale underestimates human transport/workflow constraints.
Spontaneous canine glioma models	Provide human-like brain size for realistic implant sizing and MRI-tracked biodistribution; identify device-specific complications before first-in-human studies. Limitations: cost, limited availability, heterogeneous supportive care, and ethical oversight.
Clinical translation upgrades **(e.g., RANO-PRO/QOL with correlative PK/PD sampling)**	Capture patient-centered impact and link exposure to biological response (advanced imaging, re-resection tissue, PD biomarkers). Limitations: operationally intensive but critical for distinguishing delivery failure from intrinsic resistance.

***Abbreviations:*** BBB, blood–brain barrier; CSF, cerebrospinal fluid; GBM, glioblastoma; MRI, magnetic resonance imaging; PDX, patient-derived xenograft; PK/PD, pharmacokinetics/pharmacodynamics; PRO, patient-reported outcome; QOL, quality of life; RANO, Response Assessment in Neuro-Oncology; RANO-PRO, Response Assessment in Neuro-Oncology Patient-Reported Outcomes initiative.

## Data Availability

No new data were created or analyzed in this study.

## Abbreviations

**Abbreviation**	**Definition**
μMESH	Micro-mesh
ADC	Antibody-drug conjugate
ABT-414	Depatuxizumab mafodotin
BBB	Blood–brain barrier
BCNU	Carmustine (1,3-bis[2-chloroethyl]-1-nitrosourea)
CAR	Chimeric antigen receptor
CD44	Cluster of differentiation 44
CDK4/6	Cyclin-dependent kinases 4 and 6
CED	Convection-enhanced delivery
CMC	Chemistry, manufacturing, and controls
CNS	Central nervous system
CPT-11	Irinotecan
CSF	Cerebrospinal fluid
DNA	Deoxyribonucleic acid
DNX-2401	Delta-24-RGD oncolytic adenovirus
EGFR	Epidermal growth factor receptor
FDA	U.S. Food and Drug Administration
G47Δ	Oncolytic herpes simplex virus (G47Δ)
GBM	Glioblastoma
GBO	Glioblastoma organoid
GMP	Good Manufacturing Practice
GSC	Glioblastoma stem-like cell
HA	Hyaluronic acid
HA-NP	Hyaluronic acid-nanoparticle
ICP	Intracranial pressure
IDH	Isocitrate dehydrogenase
IL13Rα2	Interleukin-13 receptor alpha 2
iRGD	Internalizing arginine-glycine-aspartic acid
LbL	Layer-by-layer
LITT	Laser interstitial thermal therapy
MAPK	Mitogen-activated protein kinase
MGMT	O^6^-methylguanine-DNA methyltransferase
MRI	Magnetic resonance imaging
mTOR	Mammalian target of rapamycin
PARP	Poly(ADP-ribose) polymerase
PD	Pharmacodynamic(s)
PDX	Patient-derived xenograft
PI3K	Phosphoinositide 3-kinase
PK/PD	Pharmacokinetics/pharmacodynamics
PRO	Patient-reported outcome
QA	Quality assurance
QOL	Quality of life
RANO	Response Assessment in Neuro-Oncology
RANO-PRO	Response Assessment in Neuro-Oncology Patient-Reported Outcomes
RAS	Rat sarcoma
RGD	Arginine-glycine-aspartic acid
RT	Radiotherapy
RTK	Receptor tyrosine kinase
TERT	Telomerase reverse transcriptase
TMZ	Temozolomide
WHO	World Health Organization

## References

[1] Louis D.N., Perry A., Wesseling P., Brat D.J., Cree I.A., Figarella-Branger D., Hawkins C., Ng H.K., Pfister S.M., Reifenberger G. (2021). The 2021 WHO Classification of Tumors of the Central Nervous System: A summary. Neuro Oncol..

[2] Stupp R., Mason W.P., van den Bent M.J., Weller M., Fisher B., Taphoorn M.J., Belanger K., Brandes A.A., Marosi C., Bogdahn U. (2005). Radiotherapy plus concomitant and adjuvant temozolomide for glioblastoma. N. Engl. J. Med..

[3] Stupp R., Taillibert S., Kanner A., Read W., Steinberg D., Lhermitte B., Toms S., Idbaih A., Ahluwalia M.S., Fink K. (2017). Effect of Tumor-Treating Fields Plus Maintenance Temozolomide vs Maintenance Temozolomide Alone on Survival in Patients With Glioblastoma: A Randomized Clinical Trial. JAMA.

[4] Kotecha R., Odia Y., Khosla A.A., Ahluwalia M.S. (2023). Key Clinical Principles in the Management of Glioblastoma. JCO Oncol. Pract..

[5] Langhans M., Popp I., Grosu A.L., Shusharina N., Binder H., Baltas D., Bortfeld T. (2023). Recurrence analysis of glioblastoma cases based on distance and dose information. Radiother. Oncol..

[6] Bastiancich C., Malfanti A., Préat V., Rahman R. (2021). Rationally designed drug delivery systems for the local treatment of resected glioblastoma. Adv. Drug Deliv. Rev..

[7] Sherriff J., Tamangani J., Senthil L., Cruickshank G., Spooner D., Jones B., Brookes C., Sanghera P. (2013). Patterns of relapse in glioblastoma multiforme following concomitant chemoradiotherapy with temozolomide. Br. J. Radiol..

[8] Zheng L., Zhou Z.R., Yu Q., Shi M., Yang Y., Zhou X., Li C., Wei Q. (2020). The Definition and Delineation of the Target Area of Radiotherapy Based on the Recurrence Pattern of Glioblastoma After Temozolomide Chemoradiotherapy. Front. Oncol..

[9] Cha G.D., Jung S., Choi S.H., Kim D.H. (2022). Local Drug Delivery Strategies for Glioblastoma Treatment. Brain Tumor Res. Treat..

[10] Gazaille C., Sicot M., Saulnier P., Eyer J., Bastiat G. (2021). Local Delivery and Glioblastoma: Why Not Combining Sustained Release and Targeting?. Front. Med. Technol..

[11] Bota D.A., Desjardins A., Quinn J.A., Affronti M.L., Friedman H.S. (2007). Interstitial chemotherapy with biodegradable BCNU (Gliadel) wafers in the treatment of malignant gliomas. Ther. Clin. Risk Manag..

[12] Brem H., Piantadosi S., Burger P.C., Walker M., Selker R., Vick N.A., Black K., Sisti M., Brem S., Mohr G. (1995). Placebo-controlled trial of safety and efficacy of intraoperative controlled delivery by biodegradable polymers of chemotherapy for recurrent gliomas. The Polymer-brain Tumor Treatment Group. Lancet.

[13] Westphal M., Hilt D.C., Bortey E., Delavault P., Olivares R., Warnke P.C., Whittle I.R., Jääskeläinen J., Ram Z. (2003). A phase 3 trial of local chemotherapy with biodegradable carmustine (BCNU) wafers (Gliadel wafers) in patients with primary malignant glioma. Neuro Oncol..

[14] Ashby L.S., Smith K.A., Stea B. (2016). Gliadel wafer implantation combined with standard radiotherapy and concurrent followed by adjuvant temozolomide for treatment of newly diagnosed high-grade glioma: A systematic literature review. World J. Surg. Oncol..

[15] Chowdhary S.A., Ryken T., Newton H.B. (2015). Survival outcomes and safety of carmustine wafers in the treatment of high-grade gliomas: A meta-analysis. J. Neurooncol..

[16] Bodell W.J., Aida T., Berger M.S., Rosenblum M.L. (1985). Repair of O^6^-(2-chloroethyl)guanine mediates the biological effects of chloroethylnitrosoureas. Environ. Health Perspect..

[17] Nikolova T., Roos W.P., Krämer O.H., Strik H.M., Kaina B. (2017). Chloroethylating nitrosoureas in cancer therapy: DNA damage, repair and cell death signaling. Biochim. Biophys. Acta Rev. Cancer.

[18] Gerson S.L., Willson J.K. (1995). O^6^-alkylguanine-DNA alkyltransferase. A target for the modulation of drug resistance. Hematol. Oncol. Clin. N. Am..

[19] Margison G.P., Povey A.C., Kaina B., Santibáñez Koref M.F. (2003). Variability and regulation of O^6^-alkylguanine-DNA alkyltransferase. Carcinogenesis.

[20] Wait S.D., Prabhu R.S., Burri S.H., Atkins T.G., Asher A.L. (2015). Polymeric drug delivery for the treatment of glioblastoma. Neuro Oncol..

[21] Hersh A.M., Gaitsch H., Alomari S., Lubelski D., Tyler B.M. (2022). Molecular Pathways and Genomic Landscape of Glioblastoma Stem Cells: Opportunities for Targeted Therapy. Cancers.

[22] Affronti M.L., Heery C.R., Herndon J.E., Rich J.N., Reardon D.A., Desjardins A., Vredenburgh J.J., Friedman A.H., Bigner D.D., Friedman H.S. (2009). Overall survival of newly diagnosed glioblastoma patients receiving carmustine wafers followed by radiation and concurrent temozolomide plus rotational multiagent chemotherapy. Cancer.

[23] McGirt M.J., Than K.D., Weingart J.D., Chaichana K.L., Attenello F.J., Olivi A., Laterra J., Kleinberg L.R., Grossman S.A., Brem H. (2009). Gliadel (BCNU) wafer plus concomitant temozolomide therapy after primary resection of glioblastoma multiforme. J. Neurosurg..

[24] Pallud J., Audureau E., Noel G., Corns R., Lechapt-Zalcman E., Duntze J., Pavlov V., Guyotat J., Hieu P.D., Le Reste P.J. (2015). Long-term results of carmustine wafer implantation for newly diagnosed glioblastomas: A controlled propensity-matched analysis of a French multicenter cohort. Neuro Oncol..

[25] Price S.J., Whittle I.R., Ashkan K., Grundy P., Cruickshank G. (2012). NICE guidance on the use of carmustine wafers in high grade gliomas: A national study on variation in practice. Br. J. Neurosurg..

[26] Weller M., van den Bent M., Preusser M., Le Rhun E., Tonn J.C., Minniti G., Bendszus M., Balana C., Chinot O., Dirven L. (2021). EANO guidelines on the diagnosis and treatment of diffuse gliomas of adulthood. Nat. Rev. Clin. Oncol..

[27] d’Avella D., DellaPuppa A. (2012). Safety and efficacy of Gliadel wafers for newly diagnosed and recurrent glioblastomas. Acta Neurochir..

[28] Mangraviti A., Tyler B., Brem H. (2015). Interstitial chemotherapy for malignant glioma: Future prospects in the era of multimodal therapy. Surg. Neurol. Int..

[29] Auffinger B., Spencer D., Pytel P., Ahmed A.U., Lesniak M.S. (2015). The role of glioma stem cells in chemotherapy resistance and glioblastoma multiforme recurrence. Expert. Rev. Neurother..

[30] Singh N., Miner A., Hennis L., Mittal S. (2021). Mechanisms of temozolomide resistance in glioblastoma—A comprehensive review. Cancer Drug Resist..

[31] Brooks L.J., Clements M.P., Burden J.J., Kocher D., Richards L., Devesa S.C., Zakka L., Woodberry M., Ellis M., Jaunmuktane Z. (2021). The white matter is a pro-differentiative niche for glioblastoma. Nat. Commun..

[32] Brooks M.D., Sengupta R., Snyder S.C., Rubin J.B. (2013). Hitting Them Where They Live: Targeting the Glioblastoma Perivascular Stem Cell Niche. Curr. Pathobiol. Rep..

[33] Fedele M., Cerchia L., Pegoraro S., Sgarra R., Manfioletti G. (2019). Proneural-Mesenchymal Transition: Phenotypic Plasticity to Acquire Multitherapy Resistance in Glioblastoma. Int. J. Mol. Sci..

[34] Wang Z., Zhang H., Xu S., Liu Z., Cheng Q. (2021). The adaptive transition of glioblastoma stem cells and its implications on treatments. Signal Transduct. Target. Ther..

[35] Dang W., Daviau T., Brem H. (1996). Morphological characterization of polyanhydride biodegradable implant gliadel during in vitro and in vivo erosion using scanning electron microscopy. Pharm. Res..

[36] Arbor Pharmaceuticals, LLC (2025). GLIADEL Wafer (Carmustine Implant) [Prescribing Information].

[37] US Food and Drug Administration (2018). GLIADEL Wafer (Carmustine Implant) [Prescribing Information].

[38] Healy A.T., Vogelbaum M.A. (2015). Convection-enhanced drug delivery for gliomas. Surg. Neurol. Int..

[39] Wang C.C., Li J., Teo C.S., Lee T. (1999). The delivery of BCNU to brain tumors. J. Control Release.

[40] Bodell W.J., Bodell A.P., Giannini D.D. (2007). Levels and distribution of BCNU in GBM tumors following intratumoral injection of DTI-015 (BCNU-ethanol). Neuro Oncol..

[41] Tabet A., Jensen M.P., Parkins C.C., Patil P.G., Watts C., Scherman O.A. (2019). Designing Next-Generation Local Drug Delivery Vehicles for Glioblastoma Adjuvant Chemotherapy: Lessons from the Clinic. Adv. Healthc. Mater..

[42] Weber E.L., Goebel E.A. (2005). Cerebral edema associated with Gliadel wafers: Two case studies. Neuro Oncol..

[43] Health Products Regulatory Authority (2025). Gliadel 7.7 mg Implant: Summary of Product Characteristics.

[44] Ricciardi L., Manini I., Cesselli D., Trungu S., Piazza A., Mangraviti A., Miscusi M., Raco A., Ius T. (2022). Carmustine Wafers Implantation in Patients With Newly Diagnosed High Grade Glioma: Is It Still an Option?. Front. Neurol..

[45] Xiao Z.Z., Wang Z.F., Lan T., Huang W.H., Zhao Y.H., Ma C., Li Z.Q. (2020). Carmustine as a Supplementary Therapeutic Option for Glioblastoma: A Systematic Review and Meta-Analysis. Front. Neurol..

[46] Dörner L., Ulmer S., Rohr A., Mehdorn H.M., Nabavi A. (2011). Space-occupying cyst development in the resection cavity of malignant gliomas following Gliadel^®^ implantation--incidence, therapeutic strategies, and outcome. J. Clin. Neurosci..

[47] McGirt M.J., Villavicencio A.T., Bulsara K.R., Friedman H.S., Friedman A.H. (2002). Management of tumor bed cysts after chemotherapeutic wafer implantation. Report of four cases. J. Neurosurg..

[48] Zhong Z., Gan L., Feng Z., Wang W., Pan X., Wu C., Huang Y. (2024). Hydrogel local drug delivery systems for postsurgical management of tumors: Status Quo and perspectives. Mater. Today Bio.

[49] Alomari S., Zhang I., Hernandez A., Kraft C.Y., Raj D., Kedda J., Tyler B. (2021). Drug Repurposing for Glioblastoma and Current Advances in Drug Delivery-A Comprehensive Review of the Literature. Biomolecules.

[50] Shapira-Furman T., Serra R., Gorelick N., Doglioli M., Tagliaferri V., Cecia A., Peters M., Kumar A., Rottenberg Y., Langer R. (2019). Biodegradable wafers releasing Temozolomide and Carmustine for the treatment of brain cancer. J. Control Release.

[51] Quinn J.A., Jiang S.X., Carter J., Reardon D.A., Desjardins A., Vredenburgh J.J., Rich J.N., Gururangan S., Friedman A.H., Bigner D.D. (2009). Phase II trial of Gliadel plus O^6^-benzylguanine in adults with recurrent glioblastoma multiforme. Clin. Cancer Res..

[52] Slika H., Shahani A., Gattu K., Mundrathi V., Solan A.A., Gonzalez B., Haque T.N., Rahman S., Sugandhi V.V., Lee J. (2025). Intracranial Nanogel Pellets Carrying Temozolomide and Paclitaxel for Adjuvant Brain Cancer Therapy. Mol. Pharm..

[53] Weingart J., Grossman S.A., Carson K.A., Fisher J.D., Delaney S.M., Rosenblum M.L., Olivi A., Judy K., Tatter S.B., Dolan M.E. (2007). Phase I trial of polifeprosan 20 with carmustine implant plus continuous infusion of intravenous O^6^-benzylguanine in adults with recurrent malignant glioma: New approaches to brain tumor therapy CNS consortium trial. J. Clin. Oncol..

[54] Zhao M., Bozzato E., Joudiou N., Ghiassinejad S., Danhier F., Gallez B., Préat V. (2019). Codelivery of paclitaxel and temozolomide through a photopolymerizable hydrogel prevents glioblastoma recurrence after surgical resection. J. Control Release.

[55] Alghamdi M., Gumbleton M., Newland B. (2021). Local delivery to malignant brain tumors: Potential biomaterial-based therapeutic/adjuvant strategies. Biomater. Sci..

[56] Bastiancich C., Danhier P., Préat V., Danhier F. (2016). Anticancer drug-loaded hydrogels as drug delivery systems for the local treatment of glioblastoma. J. Control Release.

[57] Cancer Genome Atlas Research Network (2008). Comprehensive genomic characterization defines human glioblastoma genes and core pathways. Nature.

[58] Verhaak R.G., Hoadley K.A., Purdom E., Wang V., Qi Y., Wilkerson M.D., Miller C.R., Ding L., Golub T., Mesirov J.P. (2010). Integrated genomic analysis identifies clinically relevant subtypes of glioblastoma characterized by abnormalities in PDGFRA, IDH1, EGFR, and NF1. Cancer Cell.

[59] Bow H., Hwang L.S., Schildhaus N., Xing J., Murray L., Salditch Q., Ye X., Zhang Y., Weingart J., Brem H. (2014). Local delivery of angiogenesis-inhibitor minocycline combined with radiotherapy and oral temozolomide chemotherapy in 9L glioma. J. Neurosurg..

[60] Gaitsch H., Hersh A.M., Alomari S., Tyler B.M. (2023). Dendrimer Technology in Glioma: Functional Design and Potential Applications. Cancers.

[61] Han D., Serra R., Gorelick N., Fatima U., Eberhart C.G., Brem H., Tyler B., Steckl A.J. (2019). Multi-layered core-sheath fiber membranes for controlled drug release in the local treatment of brain tumor. Sci. Rep..

[62] Li J., Xu W., Li D., Liu T., Zhang Y.S., Ding J., Chen X. (2018). Locally Deployable Nanofiber Patch for Sequential Drug Delivery in Treatment of Primary and Advanced Orthotopic Hepatomas. ACS Nano.

[63] Hersh A.M., Alomari S., Tyler B.M. (2022). Crossing the Blood-Brain Barrier: Advances in Nanoparticle Technology for Drug Delivery in Neuro-Oncology. Int. J. Mol. Sci..

[64] Bastiancich C., Bianco J., Vanvarenberg K., Ucakar B., Joudiou N., Gallez B., Bastiat G., Lagarce F., Préat V., Danhier F. (2017). Injectable nanomedicine hydrogel for local chemotherapy of glioblastoma after surgical resection. J. Control Release.

[65] Ding L., Wang Q., Shen M., Sun Y., Zhang X., Huang C., Chen J., Li R., Duan Y. (2017). Thermoresponsive nanocomposite gel for local drug delivery to suppress the growth of glioma by inducing autophagy. Autophagy.

[66] Pickering A.J., Lamson N.G., Marand M.H., Hwang W., Straehla J.P., Hammond P.T. (2023). Layer-by-Layer Polymer Functionalization Improves Nanoparticle Penetration and Glioblastoma Targeting in the Brain. ACS Nano.

[67] Shadab A., Farokhi S., Fakouri A., Mohagheghzadeh N., Noroozi A., Razavi Z.S., Karimi Rouzbahani A., Zalpoor H., Mahjoor M. (2025). Hydrogel-based nanoparticles: Revolutionizing brain tumor treatment and paving the way for future innovations. Eur. J. Med. Res..

[68] Kim J.I., Kim B., Chun C., Lee S.H., Song S.C. (2012). MRI-monitored long-term therapeutic hydrogel system for brain tumors without surgical resection. Biomaterials.

[69] Mess G., Anderson T., Kapoor S., Thombre R., Liang R., Derin E., Kempski-Leadingham K.M., Yadav S.K., Tyler B., Manbachi A. (2023). Sonodynamic Therapy for the Treatment of Glioblastoma Multiforme in a Mouse Model Using a Portable Benchtop Focused Ultrasound System. J. Vis. Exp..

[70] Yang J., Wang Z., Ma C., Tang H., Hao H., Li M., Luo X., Yang M., Gao L., Li J. (2024). Advances in Hydrogels of Drug Delivery Systems for the Local Treatment of Brain Tumors. Gels.

[71] Beola L., Iturrioz-Rodríguez N., Pucci C., Bertorelli R., Ciofani G. (2023). Drug-Loaded Lipid Magnetic Nanoparticles for Combined Local Hyperthermia and Chemotherapy against Glioblastoma Multiforme. ACS Nano.

[72] Sun Y., Chen L.G., Fan X.M., Pang J.L. (2022). Ultrasound Responsive Smart Implantable Hydrogels for Targeted Delivery of Drugs: Reviewing Current Practices. Int. J. Nanomed..

[73] Arrieta V.A., Gould A., Kim K.S., Habashy K.J., Dmello C., Vázquez-Cervantes G.I., Palacín-Aliana I., McManus G., Amidei C., Gomez C. (2024). Ultrasound-mediated delivery of doxorubicin to the brain results in immune modulation and improved responses to PD-1 blockade in gliomas. Nat. Commun..

[74] Mohammadzadeh V., Atapour-Mashhad H., Shahvali S., Salehi B., Shaban M., Shirzad M., Salahvarzi A., Mohammadi M. (2025). Hydrogels as advanced drug delivery platforms for cancer immunotherapy: Promising innovations and future outlook. J. Nanobiotechnol..

[75] Lang F.F., Conrad C., Gomez-Manzano C., Yung W.K.A., Sawaya R., Weinberg J.S., Prabhu S.S., Rao G., Fuller G.N., Aldape K.D. (2018). Phase I Study of DNX-2401 (Delta-24-RGD) Oncolytic Adenovirus: Replication and Immunotherapeutic Effects in Recurrent Malignant Glioma. J. Clin. Oncol..

[76] Todo T., Ito H., Ino Y., Ohtsu H., Ota Y., Shibahara J., Tanaka M. (2022). Intratumoral oncolytic herpes virus G47∆ for residual or recurrent glioblastoma: A phase 2 trial. Nat. Med..

[77] Nassiri F., Patil V., Yefet L.S., Singh O., Liu J., Dang R.M.A., Yamaguchi T.N., Daras M., Cloughesy T.F., Colman H. (2023). Oncolytic DNX-2401 virotherapy plus pembrolizumab in recurrent glioblastoma: A phase 1/2 trial. Nat. Med..

[78] Rui Y., Green J.J. (2021). Overcoming delivery barriers in immunotherapy for glioblastoma. Drug Deliv. Transl. Res..

[79] van Solinge T.S., Nieland L., Chiocca E.A., Broekman M.L.D. (2022). Advances in local therapy for glioblastoma-taking the fight to the tumour. Nat. Rev. Neurol..

[80] Bailly C., Vidal A., Bonnemaire C., Kraeber-Bodéré F., Chérel M., Pallardy A., Rousseau C., Garcion E., Lacoeuille F., Hindré F. (2019). Potential for Nuclear Medicine Therapy for Glioblastoma Treatment. Front. Pharmacol..

[81] Rolfe N.W., Dadario N.B., Canoll P., Bruce J.N. (2024). A Review of Therapeutic Agents Given by Convection-Enhanced Delivery for Adult Glioblastoma. Pharmaceuticals.

[82] Haddad A.F., Young J.S., Aghi M.K. (2021). Using viral vectors to deliver local immunotherapy to glioblastoma. Neurosurg. Focus.

[83] Branco F., Cunha J., Mendes M., Vitorino C., Sousa J.J. (2024). Peptide-Hitchhiking for the Development of Nanosystems in Glioblastoma. ACS Nano.

[84] Wang B., Tang D., Cui J., Jiang H., Yu J., Guo Z. (2024). RGD-based self-assembling nanodrugs for improved tumor therapy. Front. Pharmacol..

[85] Chen C., Fan R., Wang Y., Wang L., Huang C., Zhou L., Xu J., Chen H., Guo G. (2021). Hyaluronic Acid-Conjugated Nanoparticles for the Targeted Delivery of Cabazitaxel to CD44-Overexpressing Glioblastoma Cells. J. Biomed. Nanotechnol..

[86] Hayward S.L., Wilson C.L., Kidambi S. (2016). Hyaluronic acid-conjugated liposome nanoparticles for targeted delivery to CD44 overexpressing glioblastoma cells. Oncotarget.

[87] Morello A., Bianconi A., Rizzo F., Bellomo J., Meyer A.C., Garbossa D., Regli L., Cofano F. (2024). Laser Interstitial Thermotherapy (LITT) in Recurrent Glioblastoma: What Window of Opportunity for This Treatment?. Technol. Cancer Res. Treat..

[88] Lassman A.B., Pugh S.L., Wang T.J.C., Aldape K., Gan H.K., Preusser M., Vogelbaum M.A., Sulman E.P., Won M., Zhang P. (2023). Depatuxizumab mafodotin in EGFR-amplified newly diagnosed glioblastoma: A phase III randomized clinical trial. Neuro Oncol..

[89] Di Mascolo D., Guerriero I., Pesce C., Spanò R., Palange A.L., Decuzzi P. (2023). μMESH-Enabled Sustained Delivery of Molecular and Nanoformulated Drugs for Glioblastoma Treatment. ACS Nano.

[90] Bagley S.J., Logun M., Fraietta J.A., Wang X., Desai A.S., Bagley L.J., Nabavizadeh A., Jarocha D., Martins R., Maloney E. (2024). Intrathecal bivalent CAR T cells targeting EGFR and IL13Rα2 in recurrent glioblastoma: Phase 1 trial interim results. Nat. Med..

[91] Logun M., Wang X., Sun Y., Bagley S.J., Li N., Desai A., Zhang D.Y., Nasrallah M.P., Pai E.L., Oner B.S. (2025). Patient-derived glioblastoma organoids as real-time avatars for assessing responses to clinical CAR-T cell therapy. Cell Stem Cell.

[92] Kass L., Thang M., Zhang Y., DeVane C., Logan J., Tessema A., Perry J., Hingtgen S. (2024). Development of a biocompatible 3D hydrogel scaffold using continuous liquid interface production for the delivery of cell therapies to treat recurrent glioblastoma. Bioeng. Transl. Med..

[93] Ogunnaike E.A., Valdivia A., Yazdimamaghani M., Leon E., Nandi S., Hudson H., Du H., Khagi S., Gu Z., Savoldo B. (2021). Fibrin gel enhances the antitumor effects of chimeric antigen receptor T cells in glioblastoma. Sci. Adv..

[94] Tsao C.T., Kievit F.M., Ravanpay A., Erickson A.E., Jensen M.C., Ellenbogen R.G., Zhang M. (2014). Thermoreversible poly(ethylene glycol)-g-chitosan hydrogel as a therapeutic T lymphocyte depot for localized glioblastoma immunotherapy. Biomacromolecules.

[95] Buahin K.G., Brem H. (1995). Interstitial chemotherapy of experimental brain tumors: Comparison of intratumoral injection versus polymeric controlled release. J. Neurooncol..

[96] Tamargo R.J., Myseros J.S., Epstein J.I., Yang M.B., Chasin M., Brem H. (1993). Interstitial chemotherapy of the 9L gliosarcoma: Controlled release polymers for drug delivery in the brain. Cancer Res..

[97] Percie du Sert N., Hurst V., Ahluwalia A., Alam S., Avey M.T., Baker M., Browne W.J., Clark A., Cuthill I.C., Dirnagl U. (2020). The ARRIVE guidelines 2.0: Updated guidelines for reporting animal research. PLoS Biol..

[98] Jacob F., Ming G.L., Song H. (2020). Generation and biobanking of patient-derived glioblastoma organoids and their application in CAR T cell testing. Nat. Protoc..

[99] Slika H., Karimov Z., Alimonti P., Abou-Mrad T., De Fazio E., Alomari S., Tyler B. (2023). Preclinical Models and Technologies in Glioblastoma Research: Evolution, Current State, and Future Avenues. Int. J. Mol. Sci..

[100] Hubert C.G., Rivera M., Spangler L.C., Wu Q., Mack S.C., Prager B.C., Couce M., McLendon R.E., Sloan A.E., Rich J.N. (2016). A Three-Dimensional Organoid Culture System Derived from Human Glioblastomas Recapitulates the Hypoxic Gradients and Cancer Stem Cell Heterogeneity of Tumors Found In Vivo. Cancer Res..

[101] Straehla J.P., Hajal C., Safford H.C., Offeddu G.S., Boehnke N., Dacoba T.G., Wyckoff J., Kamm R.D., Hammond P.T. (2022). A predictive microfluidic model of human glioblastoma to assess trafficking of blood-brain barrier-penetrant nanoparticles. Proc. Natl. Acad. Sci. USA.

[102] Mann B., Artz N., Darawsheh R., Kram D.E., Hingtgen S., Satterlee A.B. (2025). Opportunities and challenges for patient-derived models of brain tumors in functional precision medicine. NPJ Precis. Oncol..

[103] Ratliff M., Kim H., Qi H., Kim M., Ku B., Azorin D.D., Hausmann D., Khajuria R.K., Patel A., Maier E. (2022). Patient-Derived Tumor Organoids for Guidance of Personalized Drug Therapies in Recurrent Glioblastoma. Int. J. Mol. Sci..

[104] Alcaniz J., Winkler L., Dahlmann M., Becker M., Orthmann A., Haybaeck J., Krassnig S., Skofler C., Kratzsch T., Kuhn S.A. (2023). Clinically relevant glioblastoma patient-derived xenograft models to guide drug development and identify molecular signatures. Front. Oncol..

[105] Alomari S., Kedda J., Malla A.P., Pacis V., Anastasiadis P., Xu S., McFarland E., Sukhon L., Gallo B., Rincon-Torroella J. (2022). Implementation of Minimally Invasive Brain Tumor Resection in Rodents for High Viability Tissue Collection. J. Vis. Exp..

[106] Dirven L., Armstrong T.S., Blakeley J.O., Brown P.D., Grant R., Jalali R., Leeper H., Mendoza T., Nayak L., Reijneveld J.C. (2018). Working plan for the use of patient-reported outcome measures in adults with brain tumours: A Response Assessment in Neuro-Oncology (RANO) initiative. Lancet Oncol..

[107] Scheepens J.C.C., Taphoorn M.J.B., Koekkoek J.A.F. (2024). Patient-reported outcomes in neuro-oncology. Curr. Opin. Oncol..

[108] Pelak D.M.J., Hummer A., Hug P.E., Töpfer S., Birgit Flechl I., Mozes P., Fossati P.P., Fussl C., Surböck B., Hainfellner P.J. (2025). Patient-reported outcomes, neurocognitive functioning and oncologic results of pencil-beam-scanning proton beam therapy for CNS WHO G2 and G3 IDH1-mutant diffuse adult glioma: A single institution experience. Int. J. Radiat. Oncol. Biol. Phys..

[109] Taphoorn M.J., Stupp R., Coens C., Osoba D., Kortmann R., van den Bent M.J., Mason W., Mirimanoff R.O., Baumert B.G., Eisenhauer E. (2005). Health-related quality of life in patients with glioblastoma: A randomised controlled trial. Lancet Oncol..

[110] Vera E., Christ A., Grajkowska E., Briceno N., Choi A., Crandon S.K., Wall K., Lindsley M., Leeper H.E., Levine J. (2023). Relationship between RANO-PRO Working Group standardised priority constructs and disease progression among malignant glioma patients: A retrospective cohort study. EClinicalMedicine.

[111] Noll K., King A.L., Dirven L., Armstrong T.S., Taphoorn M.J.B., Wefel J.S. (2022). Neurocognition and Health-Related Quality of Life Among Patients with Brain Tumors. Hematol. Oncol. Clin. N. Am..

[112] Dickinson P.J., LeCouteur R.A., Higgins R.J., Bringas J.R., Larson R.F., Yamashita Y., Krauze M.T., Forsayeth J., Noble C.O., Drummond D.C. (2010). Canine spontaneous glioma: A translational model system for convection-enhanced delivery. Neuro Oncol..

[113] José-López R. (2023). Chemotherapy for the treatment of intracranial glioma in dogs. Front. Vet. Sci..

[114] Hicks J., Platt S., Stewart G., Senneca C., Holmes S., Kent M., Howerth E., Kaplan J., Kaplan E. (2019). Intratumoral temozolomide in spontaneous canine gliomas: Feasibility of a novel therapy using implanted microcylinders. Vet. Med. Sci..

[115] Workman P., Aboagye E.O., Balkwill F., Balmain A., Bruder G., Chaplin D.J., Double J.A., Everitt J., Farningham D.A., Glennie M.J. (2010). Guidelines for the welfare and use of animals in cancer research. Br. J. Cancer.

[116] LeBlanc A.K., Mazcko C., Brown D.E., Koehler J.W., Miller A.D., Miller C.R., Bentley R.T., Packer R.A., Breen M., Boudreau C.E. (2016). Creation of an NCI comparative brain tumor consortium: Informing the translation of new knowledge from canine to human brain tumor patients. Neuro Oncol..

[117] Paoloni M., Khanna C. (2008). Translation of new cancer treatments from pet dogs to humans. Nat. Rev. Cancer.

[118] Spinazzi E.F., Argenziano M.G., Upadhyayula P.S., Banu M.A., Neira J.A., Higgins D.M.O., Wu P.B., Pereira B., Mahajan A., Humala N. (2022). Chronic convection-enhanced delivery of topotecan for patients with recurrent glioblastoma: A first-in-patient, single-centre, single-arm, phase 1b trial. Lancet Oncol..

[119] Upadhyayula P.S., Spinazzi E.F., Argenziano M.G., Canoll P., Bruce J.N. (2020). Convection Enhanced Delivery of Topotecan for Gliomas: A Single-Center Experience. Pharmaceutics.

